# Extradigital Glomus Tumor of Thigh

**DOI:** 10.1155/2015/638283

**Published:** 2015-07-08

**Authors:** Kemal Beksaç, Lutfi Dogan, Nazan Bozdogan, Gulay Dilek, Gokhan Giray Akgul, Cihangir Ozaslan

**Affiliations:** ^1^General Surgery Department, Dr. Abdurrahman Yurtaslan Ankara Oncology Hospital, 06200 Ankara, Turkey; ^2^Pathology Department, Dr. Abdurrahman Yurtaslan Ankara Oncology Hospital, 06200 Ankara, Turkey

## Abstract

Glomus tumors are benign neoplasms that arise from neuromyoarterial glomus bodies. They represent around 1–5% of all soft-tissue tumors. High temperature, sensitivity, and pain and localized tenderness are the classical triad of symptoms. Most glomus tumors represent in the subungual area of digits. Extradigital glomus tumors are a very rare entity. There are rare cases of these tumors reported to be in shoulder, elbow, knee, wrist, even stomach, colon, and larynx. We are reporting a case of a glomus tumor on thigh and discuss the histological and immunohistochemical features.

## 1. Introduction

Glomus tumors are benign neoplasms that arise from neuromyoarterial glomus bodies [1]. Glomus tumors represent around 1–5% of all soft-tissue tumors and 1–5% of all hand tumors [2]. They present with a classical triad of symptoms of high temperature, sensitivity, and pain and localized tenderness [3]. Most glomus tumors represent in the subungual area of digits and extradigital tumors are a rare entity. There are rare cases of these tumors reported to be in shoulder [1], elbow [4], knee [5], wrist [6], even stomach [7], colon [8], and larynx [9]. Histologically, the tumors have variable quantities of glomus cells, blood vessels, and smooth muscle cells. They are classified as solid glomus tumors, glomangiomas, and glomangiomyomas. Malignant transformation is rare. Folpe et al. propose the classification of malignant tumor as tumors with a deep location and a size of more than 2 cm or atypical mitotic figures or moderate-to-high nuclear grade and ≥5 mitotic figures/50 HPF [10]. We are reporting a case of a glomus tumor on thigh and discuss the histological and immunohistochemical features.

## 2. Case

**Figure 1:**
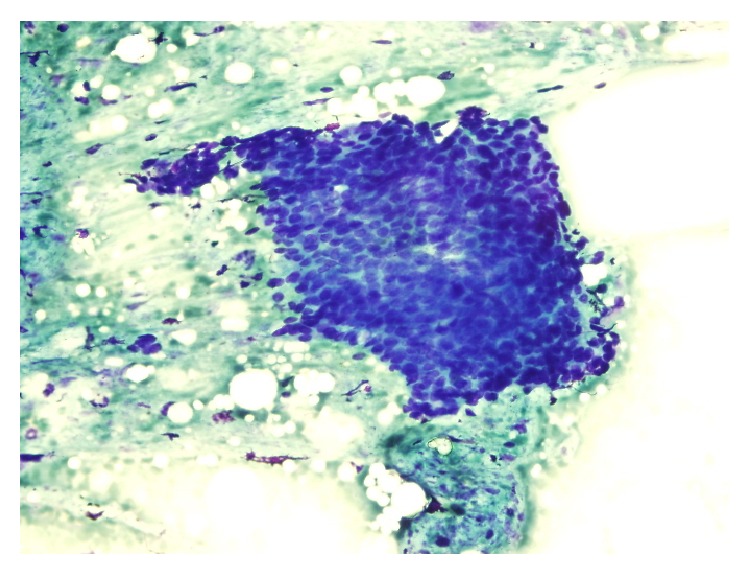
Microscopic findings of fine needle biopsy.

A 39-year-old male patient was referred to our clinic with a painful mass on his left thigh. Lesion was located on the posterolateral side and about 1/3 proximal to the head of femur. The pain had begun 2 years ago and the mass gradually increased in size during this period. The pain was moderate with a visual analogue score (VAS) 6 out of 10. Further examination with Computerized Tomography (CT) revealed a 15 × 10 mm mass in subcutaneous fat tissue. Fine needle biopsy was performed and the aspirate exhibited groups of cohesive, uniform, small, round-to-polygonal cells with scanty cytoplasm, indistinct cell borders, and round nucleus with homogeneous chromatin (Figure 1). Cells were stained with TLE-1 and this was suspicious of synovial sarcoma. Therefore lesion is excised with clear surgical margins.

Grossly lesion had a size of 1 × 1 × 1.2 cm. It was a well circumscribed blue-red nodule and had a soft consistency. In histopathologic examination, tumor was well circumscribed and encapsulated in most areas. Tumor showed diffuse or nested growth pattern. Tumor consisted of round and oval monotonous cells with pale cytoplasm and punched-out rounded nucleus. Tumor cells had no mitosis.

**Figure 2:**
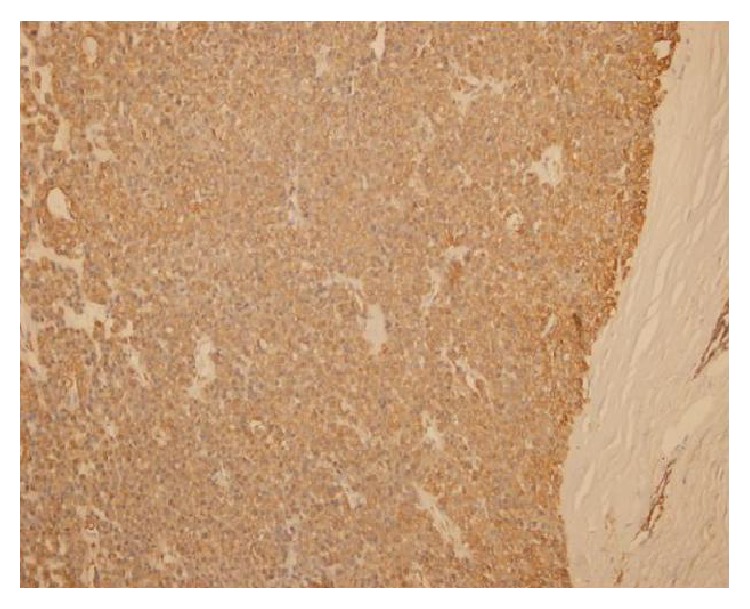
Tumoral cells staining with SMA.

**Figure 3:**
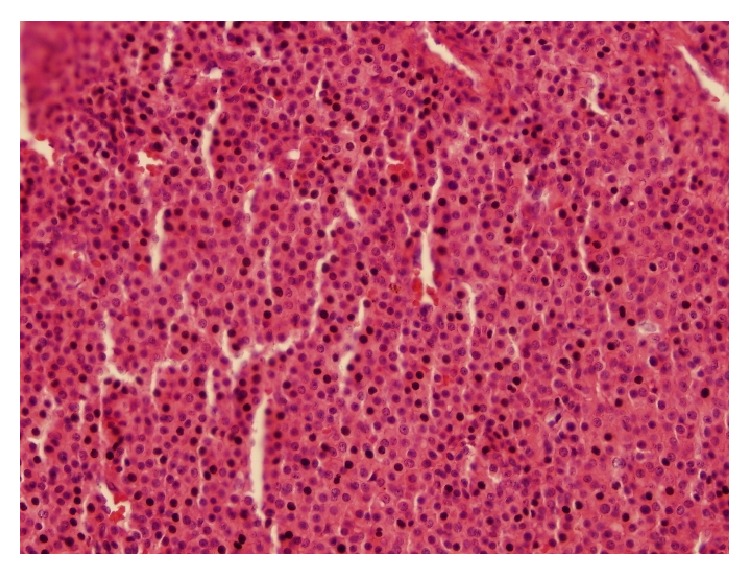
Tumor is highlighted with H&E.

In immunohistochemical examination tumoral cells stained smooth muscle actin and TLE-1. Pan cytokeratin, CD31, CD34, S-100 Fli-1, and D240 were not stained. Thus it was consistent with glomus tumor (Figures 2 and 3). No complications were observed in the postoperative period and patient was pain-free after the surgery.

## 3. Discussion

Glomus tumor is a vascular tumor believed to be originating from the cutaneous neuromyoarterial glomus body. Arteriovenous anastomoses are located between a preterminal arteriole and end efferent vein. Although most of cases originate from subungual bed of hand, there are rare cases of extradigital presentation [1, 4–9]. These tumors usually present as painful, firm, purplish, solitary subcutaneous nodules. Tumor size is generally small and rarely bigger than 1 cm. Tumors in lower extremity can have a size more than 2 cm [11]. Magnetic Resonance Imaging (MRI) is an accepted method of evaluation for subungual glomus tumors [12]. In spite of this, there are reports that even MRI is not enough to distinguish all cases [13]. Computerized Tomography (CT) is also an accepted form radiologic evaluation and especially important in the evaluation of gastric glomus tumors. It is not always possible to diagnose extremity glomus tumours with fine needle biopsy.

Treatment of choice for glomus tumor is complete surgical excision. There are also reports of alternative treatment such as sclerotherapy with sodium tetradecyl sulfate, polidocanol, and hypertonic saline and ablative therapy with argon and carbon dioxide and ethanol [14, 15].

We reported the case of an extradigital glomus tumor arising in the subcutaneous tissue of thigh. Even though glomus tumors are rare incidents and even rarer to see in extradigital locations, a review of literature suggests that these lesions may be more common than they are thought to be.
